# Seeing malaria through the eyes of affected communities: using photovoice to document local knowledge on zoonotic malaria causation and prevention practices among rural communities exposed to *Plasmodium knowlesi* malaria in Northern Borneo Island

**DOI:** 10.1186/s12936-023-04603-5

**Published:** 2023-05-26

**Authors:** Nurul Athirah Naserrudin, Pauline Pau Lin Yong, April Monroe, Richard Culleton, Sara Elizabeth Baumann, Shigeharu Sato, Rozita Hod, Mohammad Saffree Jeffree, Kamruddin Ahmed, Mohd Rohaizat Hassan

**Affiliations:** 1 Department of Community Health, Faculty of Medicine, Universiti Kebangsaan Malaysia, 56000 Kuala Lumpur, Malaysia; 2 Borneo Medical and Health Research Centre, Faculty of Medicine and Health Sciences, Universiti Malaysia, Sabah, 88400 Kota Kinabalu, Malaysia; 3 Sabah State Health Department, Ministry of Health, 88590 Kota Kinabalu, Malaysia; 4 Faculty of Social Sciences and Humanities, Universiti Malaysia Sabah, 88400 Kota Kinabalu, Malaysia; 5 Johns Hopkins Center for Communication Programs, Baltimore, MD USA; 6 Division of Molecular Parasitology, Proteo-Science Center, Ehime University, Toon, Ehime 791-0295 Japan; 7 Department of Behavioral and Community Health Sciences, University of Pittsburgh School of Public Health, Pittsburgh, PA 15261 USA; 8 Department of Public Health Medicine, Faculty of Medicine and Health Sciences, Universiti Malaysia Sabah, 88400 Kota Kinabalu, Malaysia; 9 Department of Pathology and Microbiology, Faculty of Medicine and Health Sciences, Universiti Malaysia Sabah, 88400 Kota Kinabalu, Malaysia

**Keywords:** *Plasmodium knowlesi*, Zoonotic malaria, Participatory study, Photovoice, Knowledge, Local beliefs, Preventive practises, Malaria prevention, Malaysia

## Abstract

**Background:**

Many rural communities in Malaysian Borneo and Southeast Asia are at risk of *Plasmodium knowlesi* malaria. Multiple factors contribute to infection, however, a deep understanding of illness causation and prevention practices among at-risk communities remains limited. This study aims to document local knowledge on malaria causation and preventive practices of rural communities in Sabah, Malaysia, using photovoice—a participatory research method.

**Methods:**

From January to June 2022, a photovoice study was conducted with rural communities in Matunggong subdistrict, Malaysia, to explore their experiences with and local knowledge of non-human primate malaria and prevention practices. The study included (1) an introductory phase in which participants were introduced to the photovoice method; (2) a documentation phase in which participants captured and narrated photos from their communities; (3) a discussion phase in which participants discussed photos and relevant topics through a series of three focus group discussions (FGDs) per village; and (4) a dissemination phase where selected photos were shared with key stakeholders through a photo exhibition. A purposively selected sample of 26 participants (adults > 18 years old, male, and female) from four villages participated in all phases of the study. The study activities were conducted in Sabah Malay dialect. Participants and the research team contributed to data review and analyses.

**Results:**

Rural communities in Sabah, Malaysia possess local knowledge that attributes non-human primate malaria to natural factors related to the presence of mosquitoes that bite humans and which carry “kuman-malaria” or malaria parasite. Participants revealed various preventive practises ranging from traditional practises, including burning dried leaves and using plants that produce foul odours, to non-traditional approaches such as aerosols and mosquito repellents. By engaging with researchers and policymakers, the participants or termed as co-researchers in this study, showcased their ability to learn and appreciate new knowledge and perspectives and valued the opportunity to share their voices with policymakers. The study successfully fostered a balance of power dynamics between the co-researchers, research team members and policymakers.

**Conclusion:**

There were no misconceptions about malaria causation among study participants. The insights from study participants are relevant because of their living experience with the non-human malaria. It is critical to incorporate rural community perspectives in designing locally effective and feasible malaria interventions in rural Sabah, Malaysia. Future research can consider adapting the photovoice methodology for further research with the community toward building locally tailored-malaria strategies.

**Supplementary Information:**

The online version contains supplementary material available at 10.1186/s12936-023-04603-5.

## Background

Malaria has been extensively documented across the globe in scientific journals and historical record, with evidence of the disease spanning various civilizations, including references in Hindu texts, Egyptian hieroglyphs, Chinese historical documents, and Mesopotamian texts [1]. Worldwide, there are five major species of human malaria: *Plasmodium falciparum, Plasmodium vivax, Plasmodium malariae* [2], *Plasmodium ovale curtisi* and *Plasmodium ovale wallikeri* [3]. Historically, in the Southeastt Asia region, zoonotic malaria*,* caused by the non-human primate species *Plasmodium knowlesi,* is a health threat in rural communities [4].

In 2020, the World Health Organization (WHO) included *P. knowlesi* malaria in the World Malaria Report [5]. Since the first large group of people was diagnosed with this zoonotic malaria, in Kapit, Sarawak, Malaysia [6], more cases have been detected in the neighbouring state of Sabah and in other SoutheastAsia countries [4], including in travellers [7]. Communities living or working in or near the forest are at risk, with higher cases detected among men, small-scale farmers, and large-scale agricultural workers [4]. The majority of cases are uncomplicated, but case fatality rates (CFR) were recorded at 2.5/1000 from 2015 to 2017 in Sabah, Malaysia [8]. The rate is higher in women compared to men (6.0/1000 vs. 1.7/1000) [8]. However, there were no differences in parasitaemia, and no apparent delay in health-seeking behaviour in women [8]

Since 2018, there has been no report on indigenous cases of malaria caused by the human *Plasmodium* species in Malaysia [9]. However, the increasing incidence of *P. knowlesi* malaria, ranging from 0.1 per 1000 population in Tawau, Kinabatangan, and Kota Kinabalu which are located at the East and West Coast districts in Sabah, to 1.8–3.16 per 1000 population in Keningau, an interior district and along the Crocker range from 2015 to 2017 has raised public health concerns [10]. It has been hypothesized that zoonotic spill-over of this non-human primate malaria parasite to humans is due to environmental changes and human interference with the ecology causing increasing contact between humans, macaque and the *Anopheles* mosquito vectors [11, 12]. In Sabah, the *Anopheles balabacensis* has been incriminated as the primary vector for the transmission of *P. knowlesi*, to both monkeys and humans of this zoonotic malaria [13].

Since 1960s, the malaria control programme in Malaysia has undergone progressive milestones from the eradication phase (1960–1980) to the control phase (1980–2010), to the pre-elimination phase (2011–2015), and finally to the elimination phase (2016–2020) [14]. Vector control ranges from core intervention disseminated by health personal from the Ministry of Health (MOH), such as the insecticide-treated nets (ITNs) and insecticide residual spraying (IRS) [14]. The MOH also provided supplementary interventions which includes support from health volunteers to deliver health messages and information-sharing to local communities [14]. Chua et al*.* have argued that the current vector control measures, which rely primarily on ITNs are inadequate to prevent *P. knowlesi* malaria transmission, since the *Anopheles* vector from the *Leucosphyrus* group bites outdoors after sunset [15].

Zoonotic malaria caused by species other than *P. knowlesi* has been observed in Sabah, for example, *Plasmodium cynomologi* [16]. Moreover, asymptomatic *P. knowlesi* malaria cases have occurred in the Southeast Asia region [17], including in household members who did not venture to the forest in Sabah, Malaysia [18], Indonesia [19], Myanmar [20], Thailand [21, 22], the Philippines [18], and Vietnam [23]. This emerging threat highlighted the critical need of new research methodologies, proactive strategies, and cross-sector collaboration [17].

To address this emerging threat to public health, it is crucial to explore the perspectives of affected communities. A bottom-up approach to understanding the views and perspectives of communities has the potential to increase the sustainability and impact of malaria programs [23–25]. Globally, different communities have various perspectives on illness causation [26]. For example, in the Philippines, some groups believe malaria is caused by drinking contaminated water, bathing in streams, and poor hygiene [27]. In a recent study by Iskander using photovoice, a participatory visual method (PVM), communities described body imbalances, hunger, and direct contact with dirty things as malaria risks [28]. Various communities in Africa and the indigenous “Orang Asli” population in Peninsular Malaysia, believe in supernatural causation of illness [29, 30].

Worldwide, rural communities are exposed to malaria due to social and political ecologies that influence the disease pattern [31]. For example, the low socio-economic status among indigenous communities limits their access to affordable people-centred health care and quality services [32]. Malaria interventions should consider the balance of decision-making power between various stakeholders such as the government agencies, international agencies, and communities to achieve public health impacts [24]. Although there are many studies and discussions in the literature from the researchers’ perspective, limited evidence is available from the communities' perspectives on disease transmission, specifically malaria prevention. For example, despite the state of Sabah, Malaysia having the highest *P. knowlesi* malaria cases in the world [33], understanding the issue from the perspective of communities at risk of this zoonotic disease is limited. Thus, it is crucial to engage communities to support the overall malaria strategy in the region.

The population of Sabah is culturally diverse, with more than 100 ethnicities and over 80 dialects spoken [34]. Like other malaria endemic regions, many residents in Sabah live in low socio-economic status, and low levels of formal education [35, 36]. Issues including income inequity and social and political discrimination put indigenous populations and minor ethnic groups at risk of poor health, malnourishment, and unemployment [37]. Agricultural expansion and deforestation cause ecological disturbance and macaque habitat destruction, leading to an overlapping distribution of vector mosquitoes, reservoir monkeys (*Macaca fascicularis, Macaca nemestrina* and *Presbytis melalophus*) and human settlements [12, 38]. Despite the fact that the majority of *P. knowlesi* malaria studies having been conducted in the Northern Borneo region [4], the voices of the local communities in this region are seldom heard.

To facilitate democracy of knowledge, it is essential to employ strategies that foster constructive dialogue between communities, researchers, and policymakers with regards to public health issues. According to Wallerstein et al*.* [39], adopting such methods can lead to a deeper understanding of the context and local perspectives, which could inform evidence-based policies and interventions that are more acceptable and relevant to the community encourage dialogue about public health between communities, researchers and policymakers could provide new insights based on reciprocal knowledge sharing. A study among the rural Orang Asli in Peninsular Malaysia revealed an acceptable level of knowledge and awareness of malaria; therefore, additional strategies are required to improve attitudes and practices concerning the management of malaria [30]. As such, a new research paradigm, using participatory approaches, provides space for communities to engage with research through photographs and narratives that reflect participants’ standpoints and experiences rather than just researchers' interpretations [39].

This research study aims to extend previous research in *P. knowlesi* malaria using photovoice to specifically understand local knowledge on malaria causation and the prevention practices of rural communities in Northern Borneo Island, Malaysia. Photovoice provides an opportunity to collaborate with and amplify community voices, leads to reciprocal sharing of knowledge, and empowers communities to inform policymaking through dissemination of photographs [40]. Using photovoice, this study (1) explores local knowledge on zoonotic malaria causation, (2) documents methods rural communities use to avoid mosquito bites and the reasons for their usage preferences, and (3) discusses photovoice participants’ reflections on their experience with the research.

## Methods

### Study design

The study employed a community-based participatory research (CBPR) approach, following the principle outlined by Wallerstein et al*.* [39], to ensure active participation of the affected communities in the study. This approach was particularly relevant to the photovoice methodology used in this study. The photovoice study was conducted between 1 January and 30 June 2022. The research was conducted with the collaboration of participants from rural communities in rural Borneo Island, Malaysia. These communities are exposed to *P. knowlesi* malaria [41]. Photovoice provided the opportunity to amplify community views on malaria causation and preventive practises and empower them to share the findings with policymakers [40]. Photovoice is an effective method for encouraging reflection among participants, ultimately leading to a deeper understanding of complex issues in public health.

### Study sites

Many *P. knowlesi* malaria studies have been carried out in Sabah, Malaysia [42]. Therefore, the study assumed that the community living in malaria affected areas of Sabah already has knowledge concerning malaria and the corresponding preventive practices. The study was conducted in four rural villages located in the subdistrict of Matunggong, Kudat district, Sabah state, Malaysia: Kampung (Kg.) Manduri, Kg Paradason, Kg Membatu Laut, and Kg Tagumamal Darat. This area has been the focus of numerous previous studies related with *P. knowlesi* malaria [43] and are under the surveillance of Pusat Sub Sektor (PSS) Lotong, a malaria subsector responsible for routine malaria activities.

The research team worked with the Sabah State Health Department to identify the study sites from unpublished malaria data, based on the high incidence rates of *P. knowlesi* malaria compared to other areas in the region in the year 2021. Previous studies have found *An. balabacensis* to be the primary vector for *P. knowlesi* transmission in the area [15]. The distribution of macaque monkeys in the area presents a risk for disease transmission and a potential public health threat [44]. It has also been reported that this area has a potential health burden due to the presence of asymptomatic *P. knowlesi* malaria cases in humans [16, 18].

**Fig. 1:**
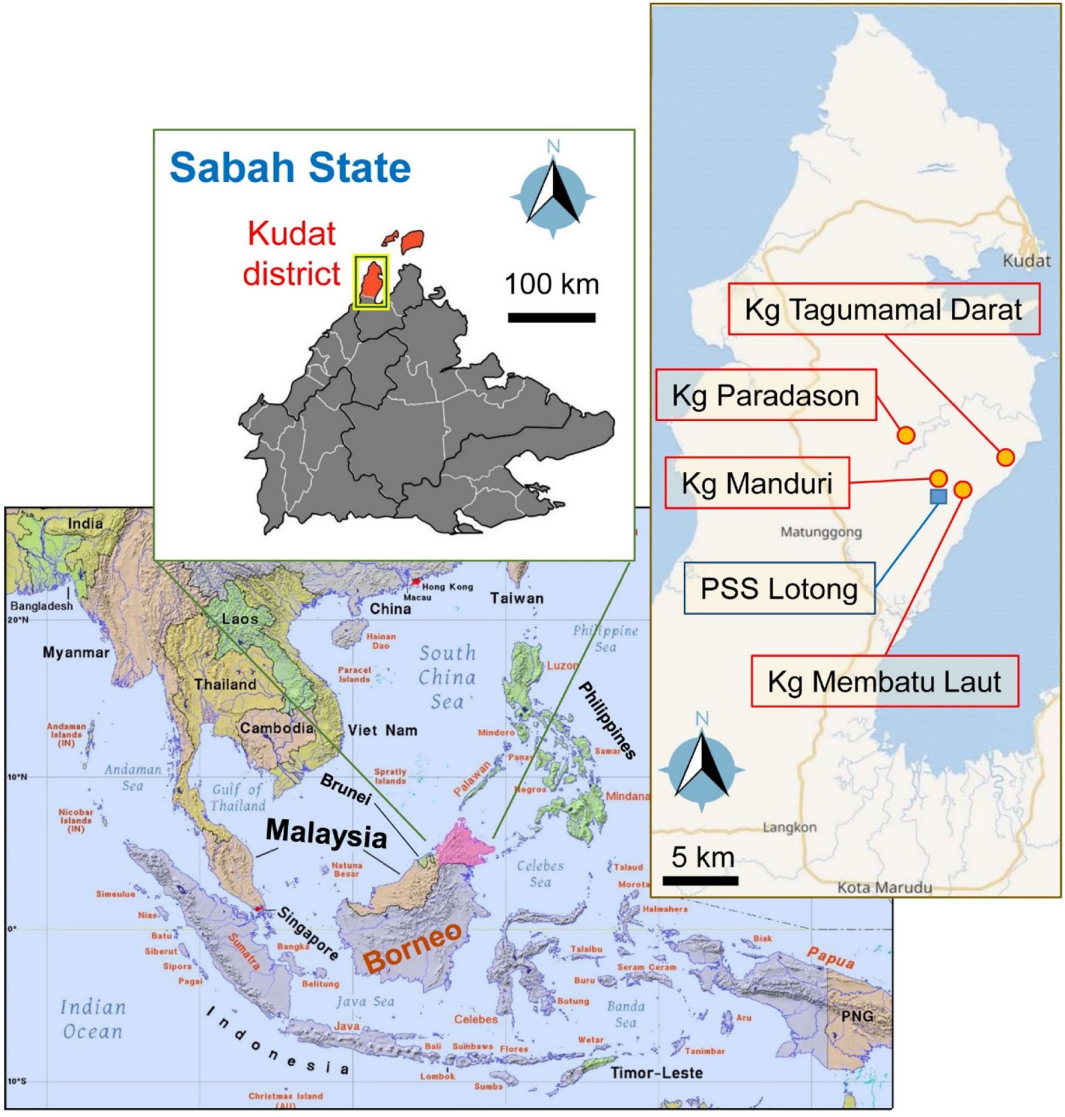
The study sites

The study sites are located 150 km from the main city of Kota Kinabalu, where the tertiary healthcare centre and diagnostic referral laboratory are located. The typical climate of the study area is tropical, characterized by two distinct monsoon seasons—the northeast monsoon from November to March and the southwest monsoon from May to September [15]. All villages shared common environmental conditions, with a wide distribution of plantations such as palm oil and rubber plantations, and forest-fringe areas [41]. The main economic sectors of the communities were centred around agriculture. However, the livelihood of the villagers mainly depended on family-plantation, fishing, paddy fields, hunting and selling forest products [44–46] (Fig. 1).

Moreover, the study area and its surrounding areas have reported other infectious diseases, which are significantly related to various socialal issues that contribute to disease occurrences. For instance, the cholera outbreak in Kudat was attributed to the lack of knowledge and access to clean water [47], while the human malaria outbreak in Kota Marudu, a neighbouring district to Kudat, was linked to theack of access to healthcare services [48]. Additionally, the Northern Borneo region is considered a tuberculosis (TB) hotspot due to various factors such as difficult access to healthcare, limited access to TB care, delayed seeking of healthcare and overcrowded informal settlements [49].

### Study participants

Photovoice is a qualitative research methodology that has been used to explore complex issues, including those related to public health [50], malaria [28], psychology [51], and marginalized populations [52, 53]. Previous photovoice studies had sample sizes ranging from 4 to 122 participants [54]. In this study, 26 participants completed all three rounds of photovoice discussions. The sample size was sufficient to generate meaningful insights into the phenomenon under study. Rather than relying on the concept of saturation, the study utilised the ‘information power’ concept proposed by Malterud et al*.* [55], which suggests that even a small number of participants can provide rich and insightful data if the study employs (i) purposive sampling (ii) has a clear research question (iii) conducts high-quality interviews, (iv) employs a rigorous analysis strategy, and (v) applies relevant underpinning theories [55]. In this study, the research team applied the logic to prioritize exploratory research, which is more powerful than research that solely aims to justify existing assumptions.

In this study, the participants acted as co-researchers as the have the first-hand experience and viewed as experts in their own lives [56]. As co-researchers, these participants engaged in the research as they participated in the data collection, analysis and dissemination of the findings.

The recruitment process for this photovoice study was facilitated by early engagement with community gatekeepers and the distribution of recruitment pamphlets in Malay language in the villages [36]. Due to the COVID-19 pandemic, and the risk of disease transmission, participants from both genders (male and female) who had fully completed COVID-19 vaccination were eligible to participate in the study. Based on the information provided by the healthcare workers (HCWs), the majority of the community members had been vaccinated. Purposive sampling methods were used for recruitment (Table 1).

**Table 1:** The eligibility criteria for study participants

Inclusion criteria	Exclusion criteria
Aged 18 years or older (at the time of data collection in 2022)	
Permanent residents of one of the four selected villages, defined as having resided in the village for at least four consecutive weeks prior to recruitment	Unable to attend and complete the photovoice discussion (whether online or in person), that could interfere with the study progress and findings, as well as those who did not remain permanently in the village due to work or educational travel to other districts
No known comorbidity associated with cognitive function or psychological issue that could impact the study progress for example during the documentation of photographs, discussion during focus group session	

### Data collection procedures

The research team consisted of a postgraduate student with over 8 years of experience in primary healthcare and public health services in rural areas of Sabah in Sabah, three epidemiologists, one medical anthropologist, one infectious disease expert, three malaria experts and one arts-based research expert. Seven members of the team have experience studying and conducting health research in Sabah, Malaysia. The research team also recruited three healthcare workers as community mediators and local translators. All team members in the field spoke the local language. The senior author conducted the meeting with gatekeepers during the study initiation to generate trust and relationship with the community leaders and HCWs [36]. The senior author completed a photovoice training workshop and held discussions with photovoice experts prior to applying the method in the field. The study adapted photovoice procedures as described in previous work [57]; (i) *Identification* of the place, person and participants, (ii) The *introduction* session was conducted in Malay Sabah dialect where participants were introduced to the study, and informed about the photography ethics, for example not taking pictures of children below 18 years old, people without shirt nor pants and other photos that are sensitive to the social norms. (iii) The *documentation and narration* phase provided the opportunity for participants to use their own smartphone to document items, human behaviours/activities, and environment and provide their own narratives of individual images that represented (a) their beliefs on how individuals or communities in their village could be exposed to malaria and (b) how they avoid mosquito bites. As part of the photovoice study, participants were asked questions such as “*How might a person contract malaria in your village*?” and “*How do you avoid mosquito bites?*”.

Participants were given 14 days to document and provide the narratives. Participants were allowed to take any number of images; overall, participants took between two to three images for each question, bringing the total to 215 images. Participants also learned about metaphoric images, where creative photos of images or environment were used to represent any ideas, people’s behaviour, place, or anything associated with their intention to inform any sensitive images, such as photos of people cleaning their body at the river or taking a shower outside their house [57]. No guidance was provided on how the picture should be taken to avoid bias towards the perceptive of the participants. (iv) The *discussion* phase was conducted, where all the focus group discussions (FGDs) sessions lasted between 60 and 90 min. The session was conducted using Malay Sabah dialect and assisted by an observer who acted as a local translator. The research team compiled the images and printed them before each FGDs discussion. The images were accessible only by the research team and saved in a password protected file. During this process, FGD notes were taken by the FGD moderator to capture key ideas during the discussion, and relevant quotes. The impressions and/or nonverbal cues were recorded by the video-recording tools to minimize the inattentiveness of the researcher with the ongoing discussion. All discussions were audio and video-recorded and saved in a password-protected device.

In total, each village held three FGDs discussions throughout the study, for a total of 12 FGDs. The first discussion explored their local understanding and knowledge of malaria causation. The second discussion focused on the communities’ preventive practices, and the third discussion focused on challenges to avoid mosquito bites and reflective thoughts throughout the discussions. During the third phase, themes generated from the interviews were confirmed with the participants. This member-checking approach increased the trustworthiness of the study findings. Field notes were written for researchers’ reflection on the study. During the FGDs, all the images were explained by participants who captured them using the SHoWeD algorithm [57].;

(i) What can we “*See*” from this photograph?
(ii) “*How*” does this relate with your life?
(iii) “*Why*” does this photograph relates with to our inquiry? For example, with malaria exposure?
(iv) “*Why*” are you using this item to avoid mosquito bites?
(v) What can be “*Done*” about the issues or challenges highlighted in this photograph?

**Fig. 2:**
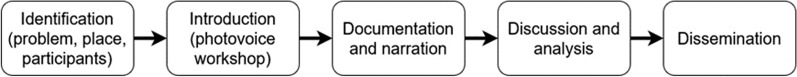
The study phases

*Analysis* was iteratively conducted during the session and after completion of the FGD. The facilitator actively listened during each FGD session, and upon completion of each session, any codes and themes that could be generated were discussed with the participants. After every FGD session, every participant received a printed image that was selected by group members that they felt best addressed the issues. All the printed images were kept in a locked cabinet accessible to the research team only. (V) During the *dissemination* phase, the images that according to the participants, addressed the issue were presented to key stakeholders, including policymakers (Fig. 2).

### Data analysis

Consistent with thematic analysis principles described by Braun and Clark [56], data analysis was iterative for this study. The senior author became familiar with the dataset by transcribing the discussions, re-reading the transcripts and generating the codes and themes from the discussions. The analysis was done in Malay language. All the study transcripts were uploaded into ATLAS.ti Windows (Version 9.1.7.0). The software facilitated the data storage and analysis. All transcripts were shared with the participants to ensure their conformability with the content and credibility (trustworthiness). This member checking technique was done after every session, including asking participants to confirm the researchers’ interpretation of the participants’ discussion and key themes. This process allows participants and the research team to participate in data analysis. The generated themes were discussed with the participants iteratively and the research team on four weekly bases while the study was underway. At the end of the study, the authors read through the transcripts before reviewing, defining the themes, and translating them into English language. The inclusion of supervision by experts increases the richness of the study findings [57]. To ensure the accuracy and credibility of the broader themes, member checking was conducted with the participants prior to the dissemination phase when sharing the study findings with policymakers. Additionally, the study findings have been reported in various sources, such as newspaper articles, online posts and reports.

## Results

In each village (Malay term as *kampung* (kg), six to eight co-researchers were recruited to participate in the documentation, narration, and analysis of the data, including the validation of themes through agreement and member checking. Overall, 22 (84.61%) of the participants were female, and the age of the co-researchers ranged between 21 and 72 years old, with a median age 34.5. In this study, seven participants (26.92%) reported a history of malaria. All participants had spouses or relatives with a history of malaria (Additional file 1: Table S1. Characteristics of study participants).

In each village, one focus group was facilitated with one moderator and one observer. The FGDs’ participants were not separated by age group or gender to allow for diverse perspectives to be exchanged in a group setting. Despite differences in gender and age, the homogeneity of the participants was characterized by social class background, language and having a similar cultural background [58, 59]. In two villages, all participants were female. In Kg. Paradason, there were two male participants. In Kg. Tagumamal Darat, there was equal participation from both genders. Unequal gender balances were mainly due to time limitation among males due to work commitments. The participants live in small villages and know each other, which helped to facilitate sharing of perspectives and experiences [59].

### Local beliefs and knowledge on monkey malaria causation

According to Murdock's theory of illness causation (1978), illness is perceived to be caused by either supernatural or natural causation [26]. In this study, the co-researchers attributed illness to natural causes, which was constructed from the physical environmental factors, such as the presence of monkeys and mosquitoes carrying the malaria parasite (referred to as “*kuman malaria*” by the community). Co-researchers also mentioned having experienced with previous research studies conducted in the village that may have contributed to the community’s awareness of monkey malaria. For example, a 55-year-old woman from Kg. Manduri stated, “*There were studies done by the White-men, from the year 2014 …until 2016….They conducted their study here too*”*.* Other co-researchers mentioned that many malaria-related activities and health messages were communicated to them in the village.

### Malaria is transmitted through the mosquito bites

All co-researchers were able to engage in discussions and share knowledge on monkey malaria exposure. One common term used in the village to describe the *Plasmodium* parasite was "*Kuman*" or "*Kuman malaria*". They reflected on the impact of continuous health program carried out by the primary healthcare clinic workers, which had helped to increase awareness of malaria in the community.

Despite the efforts to raise awareness, the co-researchers perceived a high burden of exposure to monkey malaria in the area. However, some individuals, including spouses and household members, were described as not practising the preventive measures even though they knew how malaria is transmitted. The co-researchers suggested that individual factors such as attitudes, lack of time to put on long shirts or pants, feeling hot wearing protective clothing, and ignorance could be contributing to this behaviour.

"They do understand how they can get malaria…through the mosquito bites that bring the “kuman-malaria”…but there are also people who say ‘Ah! I am strong!’ If the person already said as such-nah, that is it… maybe he or she wants to show the other person that he is strong and can avoid malaria". [Female, 33 years old, Kg Membatu Laut].

**Fig. 3:**
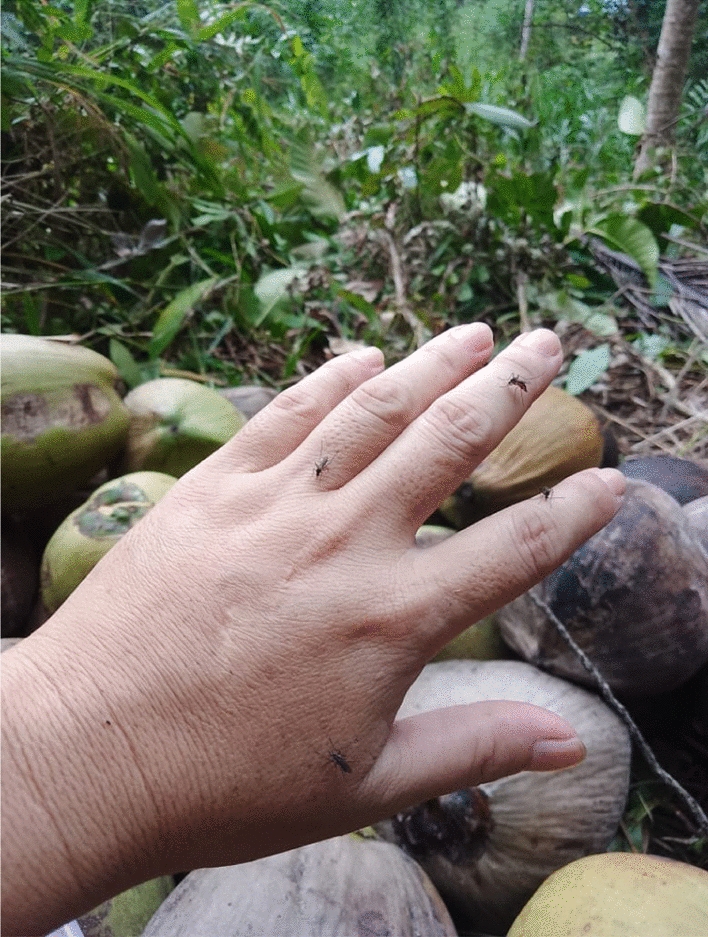
One factor a person could get malaria is a person might be unaware that he or she is being bitten by mosquitoes (Female, 49 years old, Kg Paradason)

The co-researchers were able to describe how a person can get malaria infection. Firstly, the mosquito bites the monkey, and then passes the “*kuman*” into human blood (Fig. 3).

"Sometimes we get the mosquito bites… it is very common in this village… to be exposed to them. But we do need to try to avoid them! The mosquito that brings the malaria parasite… it flies, like the rest of other mosquitoes… of course, we could not identify the mosquitoes nor knowing if the mosquito carries the parasite". [Woman, 55 years old, Kg Manduri].

**Fig. 4:**
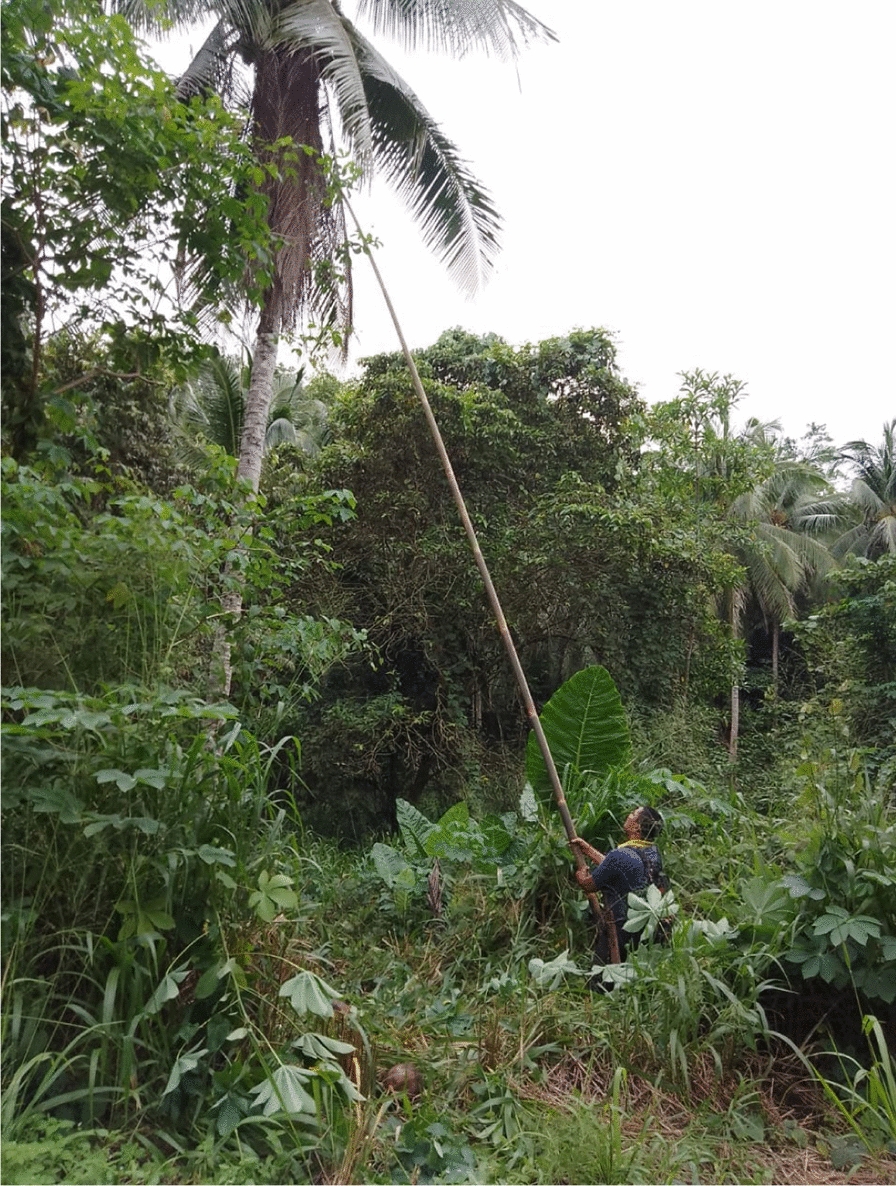
Farming activities exposed the villagers here to mosquito bites. People just wear t-shirts (Male, 41 years old, Kg Paradason)

Around the villages, there are rivers, streams, and natural ponds where children and teenagers spend time outdoors. The ponds act as a natural swimming pool for children and as natural breeding sites for mosquitoes. “*The mosquitoes like areas where there is stagnant water. They can lay their eggs there!*” [Female, 40 years old, Kg. Manduri]. The oil palm farm and plantation, water ponds also serve as breeding sites for mosquitoes. These plantations surround the village, putting community members at risk for malaria (Fig. 4).

"Over there [pointing to the oil palm plantation, coconut trees and forest in the image], even here around my house… there are many mosquitoes… there are many ponds around the villages… here [pointing to the coconut tree image], there are ponds here, and there are rivers over there… mosquitoes can breed here, there are many mosquitoes here… that is the reason people can get malaria!" [Female, 40 years old, Kg Paradason].

The community’s livelihood, lifestyle, and socio-economic status were also viewed as exposing the community to mosquito bites. During the study, co-researchers shared that activities such as gardening, gathering outside the house, chicken breeding, or family events that are commonly held in the late afternoon until night-time increased the risk of mosquito bites.

"Generally, everyone is a farmer. Those who were infected with malaria, they could have gotten the infection from their farm. For example, rubber farm, oil palm plantations… and others… so they are always at risk. From my observation, whether it is in the morning, afternoon, evening, or at night… these mosquitoes are always there, and they bite! Hahahah [laughing]. Moreover, there is a river in this village… people go there to catch fish… to do fishing activities"… [Male, 46 years old, Kg. Tagumamal Darat].

The community who are farmers, planted their vegetables such as, cassava, pumpkin, ginger, lemongrass, and others near their homes on small farms. Some individuals travel in the forest, where their farm is situated. A co-researcher whose mother had a malaria infection history will usually pick her vegetables in the evening and sell the vegetables on the next day, as early as 6 AM in the morning. Even though her mom always wears protective clothing when picking the vegetables at the farm she assumes her mother was exposed to the mosquitoes, as she will be at her stall, built with bamboo, until 6 PM until 7 PM in the evening every day.

### Rainy season and influence on mosquito breeding areas

According to the co-researchers, the weather is typically wet from December through February every year. However, when the study was conducted, there were still rainy days. One participant described the mosquitoes’ activity:

"Around 6 in the morning, when it is still breezy and humid… the mosquitos will come". [Female, 26 years old, Kg. Paradason].

Co-researchers described the risk of malaria was high in areas with mosquito breeding during the rainy season. They noted that mosquitoes were active throughout the day, with some even appearing to fly around before 6 PM in the evening:

"The mosquitoes are already flying around even before the day gets dark, even in the late afternoon. However, now, during the rainy season… there are many mosquitoes during the daytime, even in the morning… during Subuh (dawn) until 6 … 7… until the sun goes up… there are many mosquitoes during the daytime now. If it is drought season… there are no mosquitoes in the morning… because the sun is already up. But if you are at the farm… the mosquitoes are always there, no matter what time of the day". [Female, 39 years old, Kg Paradason].

During the rainy season, temporary water ponds care often created by the vehicle tracks due to the lack of gravel roads, thus increasing the risk of temporary water ponds around the villages. Co-researchers also noted that the local environment is highly conducive mosquito breeding, especially in agricultural areas such as the farm, where water can collect and create natural ponds. As one participant noted: “*When it rains, it increases our community risk to malaria… because the mosquito can breed in the water.*”

### The presence of monkeys and community exposure to monkey malaria

**Fig. 5:**
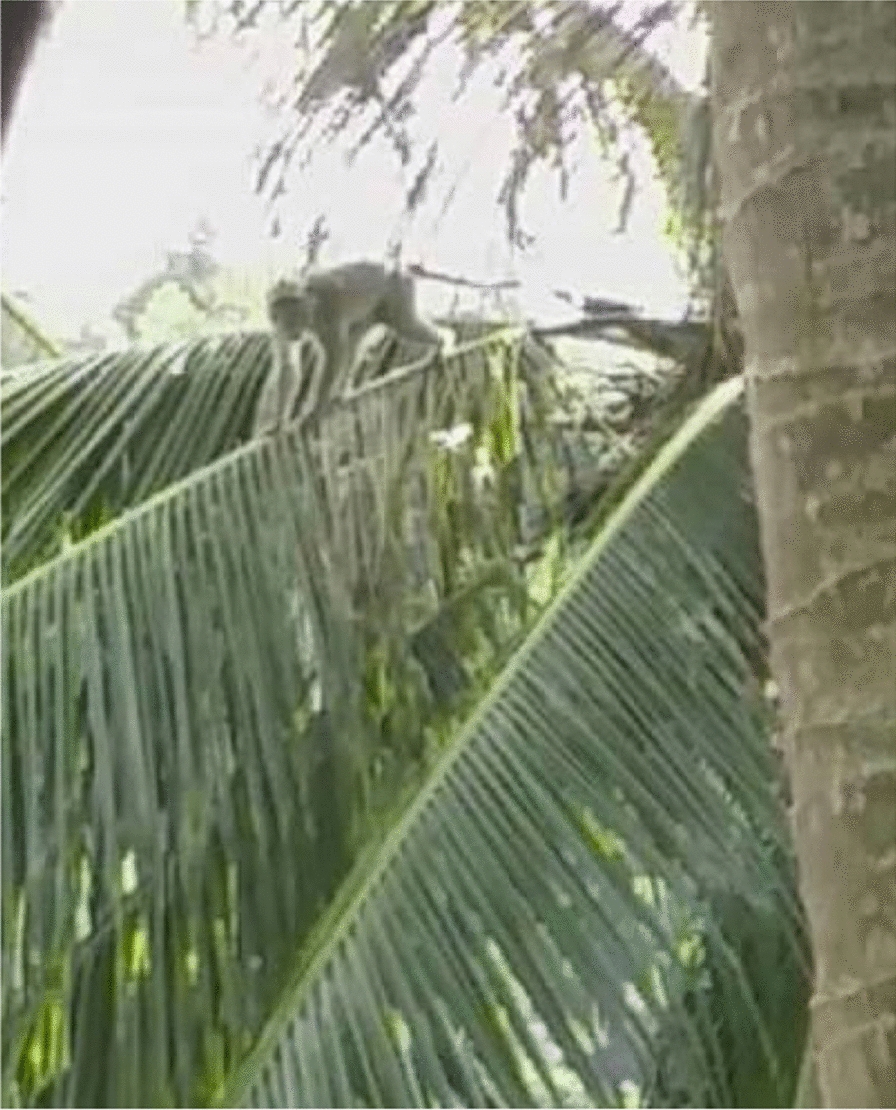
This monkey is the leader. I always see him around this area jumping from one tree to another. In his troop there are almost 50 of them. They are disturbing our farms and fruit trees! (Female, 44 years old, Kg Membatu Laut)

The village is surrounded by forest, farms and plantations that serve as a suitable habitat for mosquitoes and monkeys. The presence of the long-tailed monkeys, with skin of greyish colour, are common in their villages, unlike the other natural reservoir for *P. knowlesi* malaria such as the pig-tailed or banded-leaf monkeys. Proboscis monkeys can be found, but very rarely. The long-tailed monkeys are called ‘*kera*’ or ‘*monyet*’ (Fig. 5).

"The pig-tailed monkey, their behaviour – they are tame… but once you approach them… they will run-away… it is just their behaviour… I have participated in the previous malaria studies – done by the White-men… I went into the forest… and took pictures of the monkeys (the pig-tailed). [Woman, 55 years old, Kg Manduri]. The monkeys here, they have long tails… The pig-tailed (while looking at the pig-tailed monkey images on my phone), this one I never saw here". [Woman, 32 years old, Kg Manduri].

**Fig. 6:**
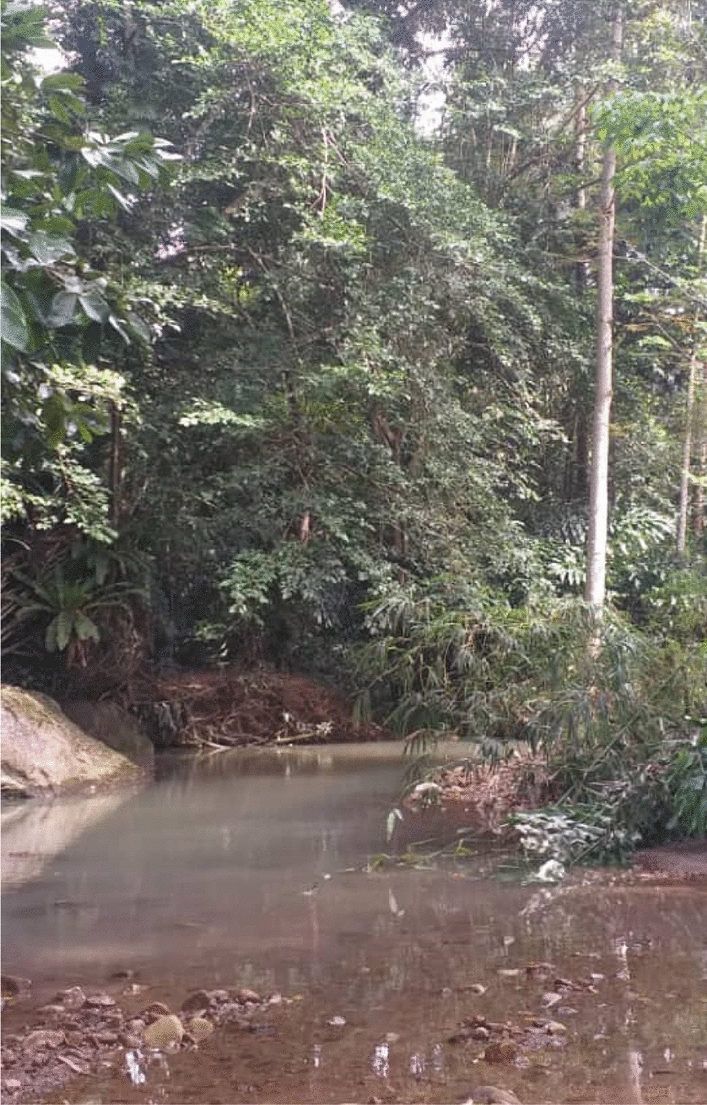
When I come here to do my laundry or clean my body, I always can see the monkey eating the “ara” fruit (Female, 44 years old, Kg Manduri)

According to the co-researchers, many fruit trees in the village such as banana, mango, coconut, and papaya trees are known to attract monkeys in the village (Fig. 6).

**Fig. 7:**
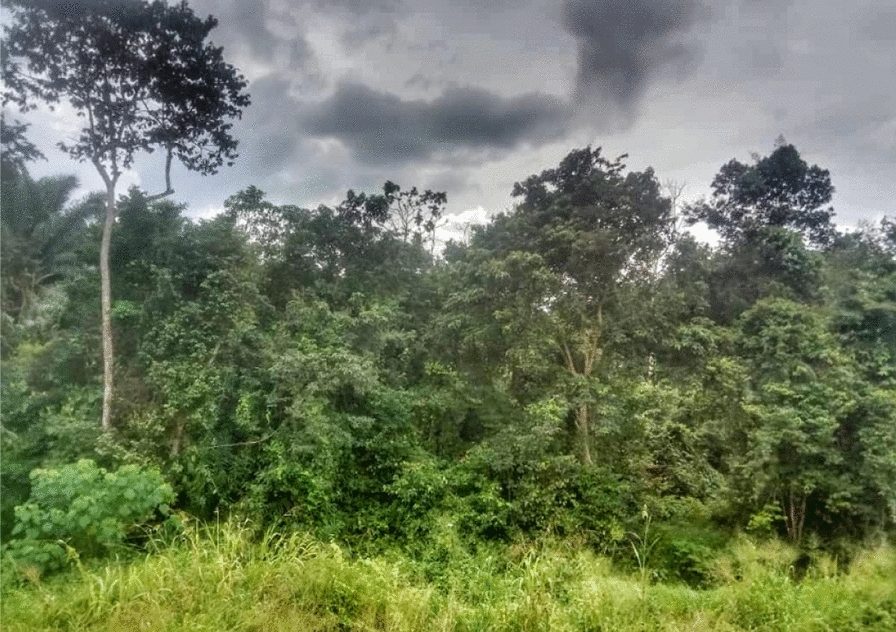
The acacia trees and the surrounding forest in our village. There are many monkeys and mosquitoes here. They exposed us to monkey malaria (Female, 49 years old, Kg Paradason)

The co-researchers reported thathuman interference has caused “damage” to the village forest. The commercial plantations of Acacia trees in the last decade disrupting the monkey’s habitat and causing damage to the villages’ surrounding. The areas have become noticeably (Fig. 7). In addition to commercial plantations like oil palm, rubber and coconut trees, villagers also practice small-scale farming on one to three hectares of land.

"Those villagers who were infected with malaria usually were exposed to the infection if they go to malaria areas. Those areas that present history of malaria cases… like the forest… and rubber tree farms… and areas with fruit trees. The monkeys will go there to eat. They like these areas … the forest habitat has been destroyed, so they come here". [Woman, 34 years old, Kg Membatu Laut].

Monkeys were not reported to be common in the last decade but are now. The co-researchers shared that the quantity of monkeys is even greater than people living in the villages and that the “never-ending” presence of mosquitoes and monkeys is disturbing their life. The monkeys adapt to human behaviour by “doing human activities,” such as eating chicken eggs, salt, and sugar in the kitchen. Monkeys even enter the villagers’ homes.

"The monkeys came into my home. They stole our salt that I put in my kitchen". [Woman, 44 years old Kg Tagumamal Darat].

The co-researchers talked about the presence of monkeys, which can be found “abundantly” in their village, and their presence is a nuisance. They disturb the farm, crops, vegetables, and paddy fields, and eat fruits such as papaya, mango, coconut, and banana, as highlighted by a female farmer from Kg. Membatu Laut: “*The monkeys… they even destroy our paddy fields!*” [Woman, 46 years old, Kg Membatu Laut].

The co-researchers noted a change in the monkeys’ behaviour, stating that they have become bold enough to enter villagers’ homes. It is believed that due to increased human activities around their village such as deforestation, commercial plantation, farming, the monkeys might have lost their natural habitat and adapted to these changes by seeking a safer area with an abundance of food, which includes human settlements. Even when shooed away from their farms or their homes, the monkeys are not easily deterred unless, they see a “senapang”(gun), which they seemed to understand as a sign of danger. Some villagers have complained to local officers, to help find a way to assist in handling the monkey disturbance, but to no avail.

### Waste disposal and risk to monkeys’ attraction and mosquito breeding sites

Co-researchers described some community members were described to practice as unhygienic methods of garbage disposal. In all the villages, there were a lack of a proper garbage disposal system, which were described as a risk factor for malaria transmission. Commonly, communities will put garbage in a designated area and burn it.

Several co-researchers highlighted the unavailability of a proper waste disposal system, which could contribute to the presence of mosquito breeding sites, such as stagnan water in tires and containers.

“Here, even near my home, many tires and containers are being dumped everywhere. It will collect the water and become a mosquito breeding site when it rains. Furthermore, the garbage disposal area, was not properly organized, and these areas became the site for mosquito breeding… there is a lot of rubbish here…monkeys goes there too, to find their food” [Female, 36 years old, Kg. Membatu Laut].

Moreover, co-researchers reported that with no proper area for garbage disposal, there is a risk of localities harbouring continuous breeding sites for mosquitoes. They added, the hygiene of the village affects the cleanliness and health of the neighbourhood, that when the garbages are not tended to, they become a source for mosquito breeding sites.

### Avoiding mosquito bites

**Fig. 8:**
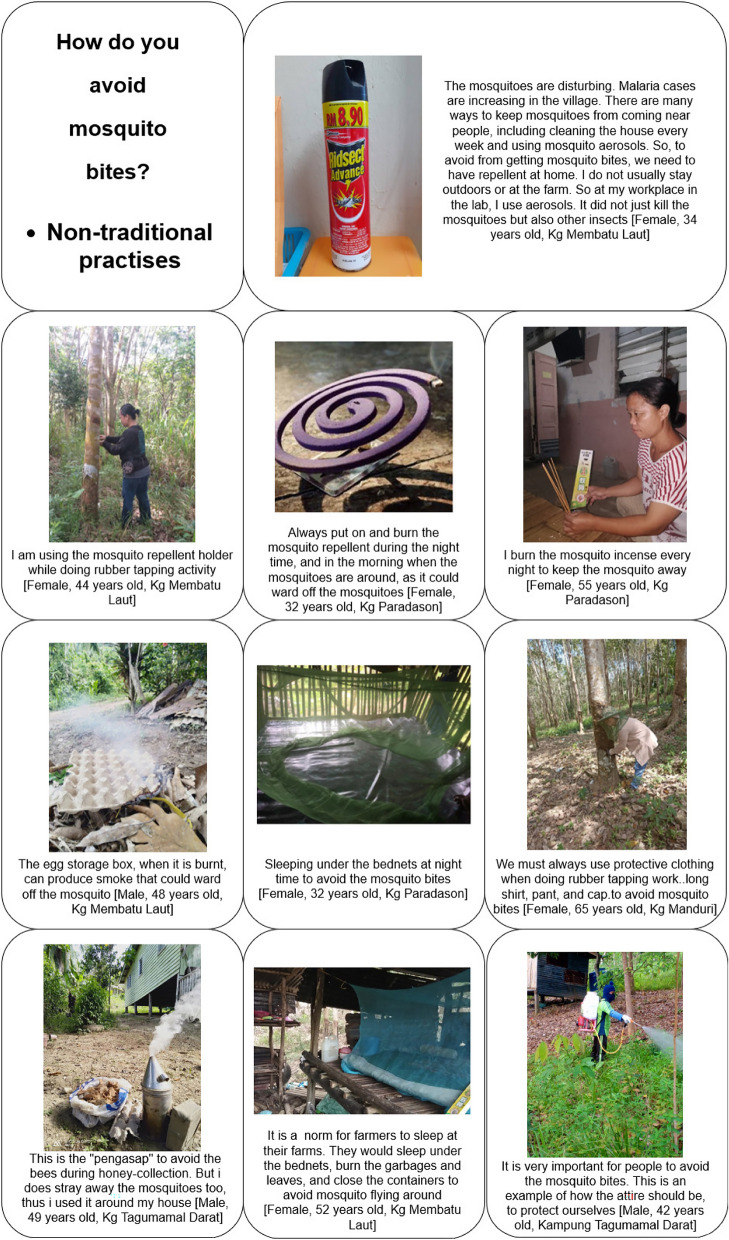
Non-traditional practices for preventing mosquito bites

**Fig. 9:**
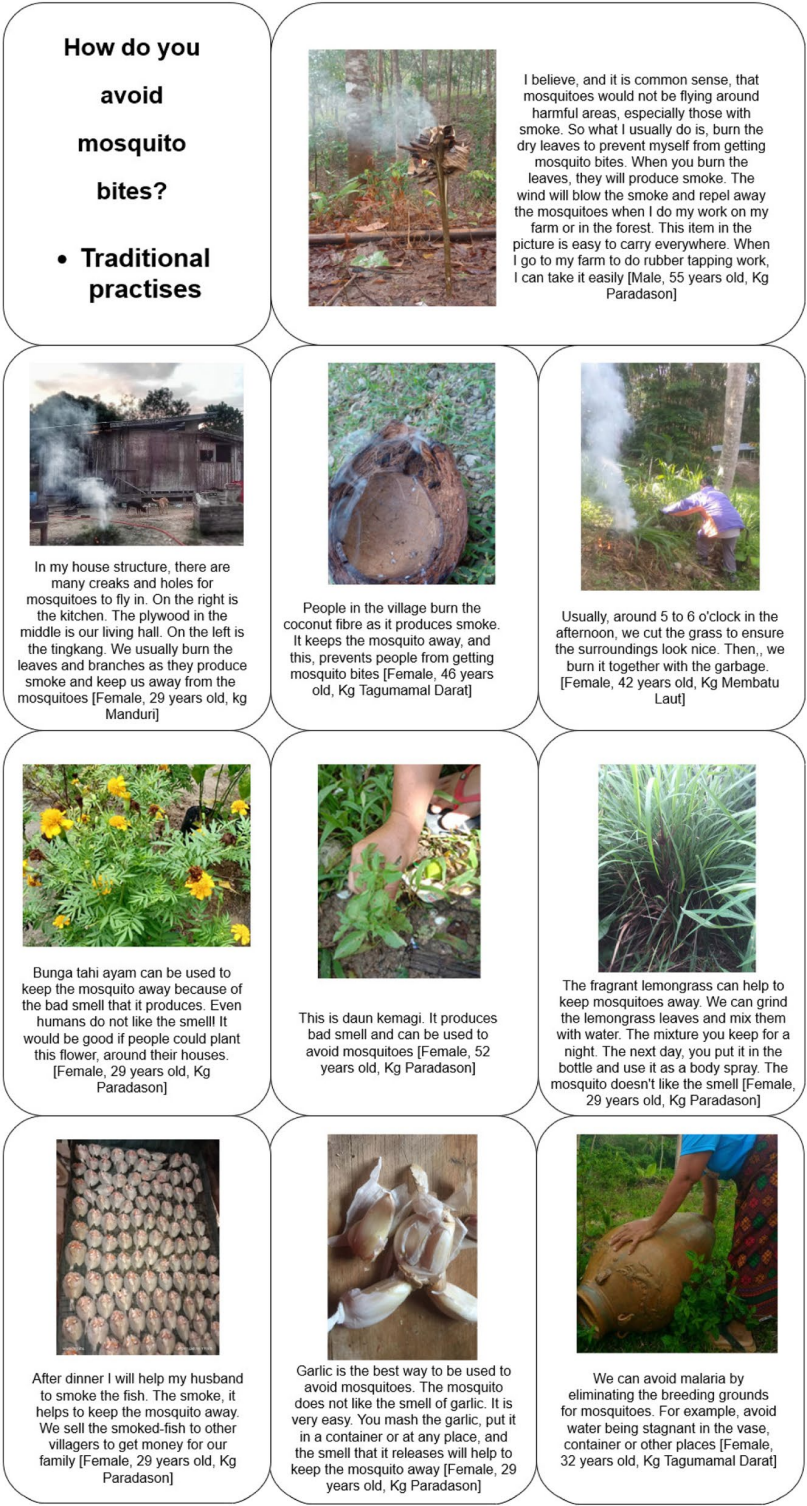
Non-traditional practices for preventing mosquito bites

Co-researchers described several preventive practices to avoid mosquito bites. These practices were grouped into two categories, non-traditional practises (Fig. 8), which were defined as factory-produced products designed to prevent mosquito bites, and traditional practices (Fig. 9).

### Traditional practices

According to the co-researchers, traditional practices for repelling mosquitoes are popular among the community due to their sustainable and cost-free nature. They reported, community members using a variety of natural materials, such as coconut fibre, leaves, and branches, to produce smoke that could ward off mosquitoes during outdoor activities:

“I used coconut, I burnt them, it produces smoke… this is one way we use natural products to avoid mosquitoes in the village… like myself, when I want to relax with my coffee, before the sunset… I burn the coconut fibre…” [Female, 33 years old, Kg Membatu laut].

In Kg. Paradason, co-researchers described how, after-fishing, their husbands would smoke the fish at night-time, and the resulting smoke would help to ward off mosquitoes.

Additionally, they mentioned the use of such as bunga tahi ayam (*Lantana camara*), Serai wangi (lemongrass, *Cymbopogon nardus*), Daun kemangi (Thai basil, *Ocimum basilicum*), *tuhau* (local wild edible ginger) and garlic, which produce smells that mosquitoes find unappealing, “Bunga tahi ayam”, fragrant lemongrass and Thai basil are planted by several co-researchers and community members around their house. These plants are readily available in the village and can be easily planted and maintained.

Co-researchers shared various ways to use natural plants to repel mosquitoes. For instance, one co-researcher shared that the lemongrass could be crushed, producing a watery substance that could be applied on the skin, while another explained that the garlic can be creatively worn as an accessory around the waist or neck, or crushed and placed aroundthe house to ward off mosquitoes.

“When I was in school, one of the teachers taught us about these plants… our science teacher, from Peninsular Malaysia… taught us a lot about the plants that could ward off mosquitoes… these plants, they have a unique smell… for example, the bunga tahi ayam, serai wangi… tuhau. Do you know that we use garlic… it is the best way to protect ourselves from mosquitoes… they don’t like the smell. It is easy, you crush them… put them in a plate or box so that the smell is released… the mosquitoes will fly away from the area.” [Female, 24 years old, Kg. Paradason].

Furthermore, co-researchers emphasized the importance preventing stagnant water around the house and in the villages, as well as the effectiveness of using hands to kill mosquitoes:

“If I am lazy to burn the leaves, or colok, naaaa… we do like this (flipping the cloth around her body, acting the way she wards off the mosquitoes). When I am outside, and do my work, like rubber tapping… I take off my shawl or “kain lap”, I do like this (flipping the shawl) … so that the mosquitoes do not come near me. These mosquitoes, they are light animals, they fly, once the shawl hits them, they will die.” [Female, 40 years old, Kg Paradason].

These traditional practices are accessible to all community members, regardless of their socio-economic status, and according to the co-researchers making them a sustainable and cost-effective alternatives to non-traditional approaches to avoid mosquitoes.

### Non-traditional practices

Many co-researchers reported that the non-traditional (modern) items are (1) not feasible for all occasions, (2) are unaffordable (3) cause discomfort, in particular breathing difficulty and eye irritation (4) are not functional if used outdoors, and (5) do not kill the mosquitoes. Items such as mosquito incense and repellents, while viewed as helping to ward off mosquitoes, were viewed as too expensive to use daily, or for several occasions. When used outdoors, it cannot ward off mosquitoes as the smoke that was produced flows accordingly with the wind. Full coverage was not possible with these items.

“The colok (English; mosquito incense), I use them when I am inside the house… this colok is good if it is used indoor, but not during outdoor activities. Moreover, it burns off quickly. If I use colok, the smoke does not cover the whole area… it is just there… it burns off quickly… the mosquitoes will not die… they will not die.” [Female, 33 years old, Kg Membatu Laut].

Some co-researchers described that mosquito incense, repellent, and aerosols as expensive products. Villagers could not afford to purchase them: “*The colok, it is expensive… one box will cost you RM2.50… If I buy five boxes, it cost me RM10.00.*” [Female, 24 years old, Kg Paradason].

Co-researchers also shared the benefits of using ITNs, as evidenced by the story of a 100-year-old lady who never contracted malaria because she always used a bed net while sleeping. The granddaughter who participated in the study quoted: “[*While pointing to the image of her grandmother and her bed net*]* This is my grandmother, she always put on the bed nets during sleep. After all these years, she was never infected with malaria. She is almost 100 years old, maybe more… she never forgot to put on the bed nets during sleep.*” [Female, 36 years old, Kg Membatu Laut].

Another effective item used by the co-researchers was the “alat pengasap ketika mengambil madu” (English: bee smoker), which was provided exclusively to those who enrolled in honey-farming activities. During honey collection, the bee smoker produced smoke that not only ward off bees but also mosquitoes. As individual collected honey from the hive, they carried the smoker to keep the bees away and, indirectly, ward off mosquitoes as well. One participant a 53-year-old male from Kg Tagumamal Darat, engaged in a variety of activities such as chicken breeding, rubber tapping, and oil palm plantation work but was never diagnosed with malaria.

A female from Kg. Paradason shared that she has never been infected with malaria despite engaging in various outdoor activities. She believed that her success in avoiding mosquito bites to the use of protective clothing, including covering the head and hands. Another female from Kg. Paradason also shared a similar experience, noting that wearing protective clothing has helped her avoid malaria. She stated: “*If I go out of my home – to have a walk… I will wear a long shirt and pants.*” [Female, 24 years old, Kg. Paradason].”

### Photovoice participant reflections

The participants, who were recruited from the local community, shared how the project impacted their perspective on knowledge creation through the use of the photovoice methodology. The collective opinion sharing through discussions enabled them to learn from their peers and the research team. These co-researchers emphasized how photovoice allowed them to recognize issues from the perspectives of other c-researchers and appreciate the diversity of viewpoints. They also described the experience as empowering for themselves:

“People in the village were curious…. Why do I go around in the village snapping pictures using my phone. I told them, I am doing research, I am a researcher now…When I went around the village, and I saw areas with rubbish, I felt sad, I felt miserable. Thus, I cleaned them…. To avoid mosquito breeding sites.” [Female, 45 years old, Kg Membatu Laut.

**Fig. 10:**
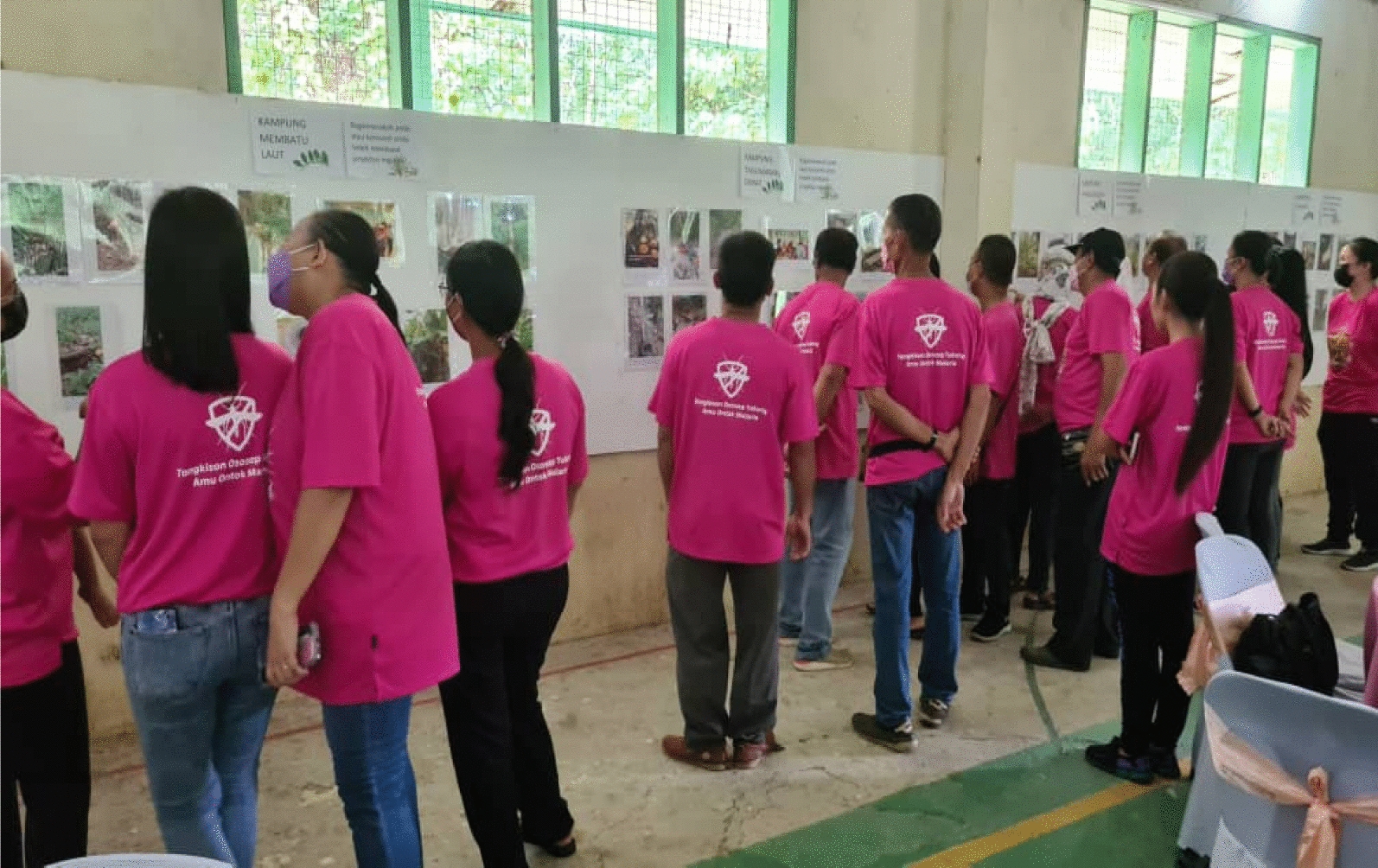
The photo exhibition in Kudat, Sabah, Malaysia

Co-researchers gained new knowledge through participating in the study: “*I never knew previously that the serai wangi (English; fragrant lemongrass) could ward off the mosquitoes!*” [Female, 33 years old, Kg Membatu laut]. In Kg. Tagumamal Darat, not all co-researchers knew that *tuhau* could ward off mosquitoes. Commonly, *tuhau* was only served as part of their meal: “*I never knew this plant could ward off mosquitoes… I only cook and eat them*!” [Female, 34 years old, Kg Tagumamal Darat] “. The co-researchers appreciated the reciprocal sharing of knowledge:

“I saw other participants shared their images, and everyone gave their own perspectives, and we can discuss about it during the session… I learn about malaria; I learn about how other people avoid the mosquitoes… I did not go to secondary school… I appreciate all this new knowledge.” [Female, 45 years old, Kg Membatu Laut (Fig. 10).

Further, they described their experiences during the photo exhibition. The photo exhibitions facilitated conversations about the beliefs, local knowledge and how the community avoided mosquito bites using various measures/items. Attendees had the opportunity to engage with the research team, other photovoice participants, community members and policymakers such as the officers from MOH and academicians. The photovoice study empowered co-researchers to provide narratives fo the images taken and allowed their voices to be heard, communicating their challenges to policymakers. During the exhibition, c-researchers could address questions raised by the stakeholders. Co-researchers also were able to share their views during the experience sharing session:

“The monkeys, they disturb our farm, our plants, fruit trees… we hope that someone could find a way. Killing them is inhumane… but if they could be shifted to other areas, that would be highly appreciated.” [Male, 68 years old, Kg Tagumamal Darat].

Although previous research has been conducted on this community, the current study was unique as it was conducted in partnership with the participants who were recruited as co-researchers. During the exhibition, one co-researcher shared their hope:

“The mosquitoes, they are always here in the village… we hope any organization or anyone, could help to find ways to avoid the mosquitoes. This is important because most of us are farmers, we need to work, we need to go to the farm… we need to go for rubber tapping. It is our livelihood… the way that we could get money.” [Female, 55 years old, Kg Manduri].

The comments from co-researchers suggest that photovoice worked against the silence around malaria prevention and helped them change their perspective on their vulnerability to malaria. The study initiated critical reflection among co-reseachers and served as a platform for initiating policy change:

“So, the relevant governance should hear us out… so we could deal with this issue together… we can help each other to reduce the malaria cases here.” [Male, 46 years old, Kg. Tagumamal Darat].

The CBPR value of photovoice was deeply evident in this work. Although the impact and policy change were not identified in this study, the photo exhibition offered a platform that emphasized the reciprocal sharing of knowledge, raising awareness, and lobbying innovative ways for malaria prevention strategies. The exhibition directly messages and engages attendees, and presents the issues faced by communities. The study helped co-researchers change how they think about their vulnerability to malaria. Clearly, this study initiated critical reflection among co-researchers [60] and served as a platform for initiating policy change.

## Discussion

This is the first study to explore the perspectives of rural communities in Malaysian Borneo concerning their beliefs and knowledge of malaria causation and associated preventive practices using the photovoice methodology. Despite the challenges posed by COVID-19 pandemic, the photovoice study proved effective in exploring health issues related to zoonotic malaria and was well-received by the communities, as evidenced by their willingness to participate and share their photographs. Notably, the study found no instances of misinformation on how a person can get malaria. The co-researchers demonstrated a clear understanding of the natural causes of zoonotic malaria, transmitted through mosquito bites from infected monkeys. Nonetheless, the study revealed new understanding of the risks of malaria in these communities.

The co-researchers reported the presence of mosquitoes early during the day in the villages. Previous studies have indicated that the major vector of *P. knowlesi* in this region, *An. balabacensis*, bites at dusk [13, 15]. *Anopheles balabacensis* is simio-anthropophilic in nature [61]. Despite this discrepancy, the villagers' livelihoods, lifestyle and socio-economic activities expose them zoonotic malaria, which poses a potential threat to the malaria elimination programme in Malaysia.

The study highlights the financial constraints that prevent households in Kudat, one of the poorest communities in the state, from purchasing mosquito control products. Given the low socio-economic status, it is unlikely that households can allocate significant funds for anti-mosquito measures. To build on these findings, the malaria control programme may consider extending this study to other locations to verify the findings. Moreover, dialogues with the community are needed to co-create strategies and improve health programmes.

The study highlights the effectiveness of the photovoice methodology in identifying the diverse range of malaria prevention practices in rural communities in Sabah, Malaysia, including traditional methods that use local plants to repel mosquitoes to non-traditional practices such as burning various mosquito products to produce smoke and the usage of ‘*alat pengasap ketika mengambil madu*”(madu” (English: bee smoker). The cost-effectiveness of traditional practices make them a preferred option in these communities. Additionally, plant-based repellents are considered better for the environment than synthetic molecules and have been used for generations as a personal protection measure against mosquitoes [62].

Plant-based repellents are still extensively used in this traditional way throughout rural communities in the tropics because, for many of the poorest communities, they are the only means of protection from mosquito bites [62, 63]. Despite their effectiveness, traditional methods may not be enough to achieve the goal of malaria elimination by 2030. A collaborative effort between different sectors, including, pharmaceutical companies, and local communities, could bridge the gap and develop more effective measures. Photovoice could help process the findings as new information to policymakers [55], raise questions about more effective interventions and provide an open platform to communities, policymakers, and other researchers towards a more collaborative effort in disease control.

Previous photovoice studies have informed policy change on various health issues such as HIV/AIDS prevention, healthy food choices, and family planning [54]. Similarly, policymakers and must support more effective, affordable, and feasible vector control measures in rural communities to achieve the malaria elimination target.

Through the photo exhibition, co-researchers were invited to voice their perceptions and experiences to a broader audience, including policymakers, thus initiating community empowerment. Community leaders and policymakers were invited to the photo exhibition, allowing the co-researchers to share their experiences and ideas for health equity, social change, and action [40]. The challenges faced by the communities to avoid malaria were the main highlight. The event created space for future prospects and continued collaboration between participants, research team and stakeholders. Future studies should look into expanding the context of co-designing interventions with at-risk communities towards a more emancipatory approach [39]. These transformative approaches bridge the gap between communities and policymakers and serve as a platform to guide strategy for the control and elimination of malaria. Future studies could adapt the study methodology and/or findings to escalate research priorities, adapt the relevant preventive practices and improve malaria control. Public health researchers can adapt photovoice methodology to address social-related contexts concerning other health issues.

### Limitations

The FGDs faced challenges of dominant group members [58, 59], which may have influenced the perspectives shared by the participants. However, researchers minimized power dynamics between genders and age by allowing all participants to share their perspectives and prompting non-dominant individuals to participants in the discussion. The moderator prompted the non-dominant (silent) individuals by “breaking the ice” with older group members (or men), and stimulated the discussion afterward.

The sample size and sampling approach were selected for richness of information, but may not produce broadly generalizable results.

A limited number of policymakers were invited during the exhibition due to insufficient resources for the event. Despite these limitations, the study created a collaborative partnership and reciprocal knowledge-sharing with the participants. Limited funding also prevented the photo exhibition from being conducted in additional locations such as the main city, district or state. Therefore, the research team compensated for this challenge by acknowledging the participation of community members in academic manuscripts, conference presentations, and other platforms.

### Strengths

The photovoice study findings allowed for a better understanding of issues more accurately through the perspective of local communities and create a collaborative partnership with the participants. The ability to practice cultural humility among the research team allowed genuine participation throughout the research process, including introduction workshop, dialogue or discussion session and dissemination of the findings. The FGDs encouraged participants to freely express their views within the group, fostering open communication.

### Future recommendations

The study strongly resonated with the participants as they were able to share their voices, concerns, and challenges. As the photovoice study allowed the integration of photos and narratives by the local communities and the ability to create critical dialogues with participants, the study could be expanded to other areas to better understand issues related to *P. knowlesi* malaria. Photovoice could address the issues and challenges of avoiding mosquito bites among rural communities. A continuous engagement with participants could allow for opportunities to share information jointly with policymakers. The partnership created in in this study, could incorporate political action towards advancing social equity [39]. Future prospects should look into studies and strategies that could facilitate in designing of locally targeted malaria interventions and evaluations.

Regarding the dissemination of the findings, in addition to the photo exhibition, the findings will be shared through different platforms for example peer review articles and a photovoice book. Across these different platforms, it could help to provide new insights in controlling zoonotic malaria in rural communities. Due to the limited time and resources during the exhibition, future dialogues and discussions could be done with policymakers, stakeholders, and other sectors to share the findings in more detail to identify actionable solutions to limitations, challenges, and community concerns. In the effort to control zoonotic malaria, the participation of communities in research and disease control strategies is critical.

## Conclusion

This photovoice study revealed no misconceptions on malaria causation among participants living in rural villages in Sabah, Malaysia. The study provided valuable information and new insights concerning the various practices that are used to avoid mosquito bites, which are influenced by factors such as cost, feasibility, and availability of interventions. These findings underscore the importance of developing locally targeted vector control measures that are based on the needs and preferences of the communities affected by *P. knowlesi* malaria. Moving forward, multi-sectoral collaboration will be crucial in implementing effective and sustainable strategies for malaria control in these areas.

## Supplementary Information

**Additional file 1: Table S1.** Summary description of participants.

## Data Availability

All data used and/or analysed during this study are available upon request to the corresponding author.

## Abbreviations

CFR: Case fatality rates
PVM: Participatory visual method
CBPR: Community-based participatory research
Kg: Kampung
PSS: Pusat sub sector
TB: Tuberculosis
HCWs: Healthcare workers
ITNs: Insecticide-treated nets.
IRS: Insecticide residual spraying
FGDs: Focus group discussions
